# Beyond the lab: Eh!woza and knowing tuberculosis

**DOI:** 10.1136/medhum-2018-011479

**Published:** 2018-11-27

**Authors:** Bianca Masuku, Nolwazi Mkhwanazi, Ed Young, Anastasia Koch, Digby Warner

**Affiliations:** 1 SAMRC/NHLS/UCT Molecular Mycobacteriology Research Unit, Department of Pathology, Institute of Infectious Diseases and Molecular Medicine, Faculty of Health Sciences, University of Cape Town, Cape Town, South Africa; 2 Wits Institute for Social and Economic Research (WiSER), University of the Witwatersrand, Johannesburg, South Africa; 3 Wellcome Center for Infectious Diseases Research in Africa, Institute of Infectious Diseases and Molecular Medicine, Faculty of Health Sciences, University of Cape Town, Cape Town, Western Cape, South Africa

**Keywords:** TB, biomedicine, youth, knowledge, public engagement, film

## Abstract

Eh!woza is a public engagement initiative that explores the biomedical and social aspects of tuberculosis (TB) in South Africa. The project is a collaboration between scientists based in an infectious disease research institute, a local conceptual/visual artist, a youth-based educational non-governmental organization (NGO) and young learners from a high-burden TB community. The learners participate in a series of interactive science and media production workshops: initially presented with biomedical knowledge about TB and, in later sessions, are trained in creating documentary films and engage with ideas around visual representation. The participants are encouraged to make use of this newly acquired knowledge to tell stories from their chosen communities in Khayelitsha, a township in Cape Town. Through its engagement with the complex manner in which TB is experienced, framed and understood by biomedical scientists, young people, and those who have been affected by the disease, Eh!woza presents alternative ways of exploring the complexities of human illness. The integration and interrogation of biomedical understandings, lay narratives and the young participants’ framing of the disease poses questions about ’knowing', and the meanings people attribute to ways of ’knowing' and the actions they impel. The project also presents contrasting reflections on cure—from a biomedical perspective, and care—from the perspective of TB-affected young people and community members. In this article, we describe the Eh!woza project, present thoughts from the participating students on the science and media workshops, and detail the narratives of ill-health and disease from people within their neighbourhoods. We conclude with a critical analysis of the complexities of knowledge communication, notions of cure versus care, and a consideration of the potential contribution of this project to the growth of medical humanities in Africa.

## Introduction

In 1993, WHO declared tuberculosis (TB) a ‘global emergency’. Almost three decades later—and on the eve of the first ever high-level meeting of the United Nations General Assembly on TB, scheduled for 26 September 2018 and themed ‘United to end tuberculosis: an urgent global response to a global epidemic’—several hundred thousand people in South Africa continue to contract TB every year, with the country consistently ranking among the highest of those with rates of TB-related morbidity and mortality globally.^1^ In addition to the significant burden on the public health system, TB is often accompanied by severe economic and social consequences—a situation exacerbated by the co-epidemic of HIV/AIDS and the emergence of drug-resistant TB.^2^

In South Africa, research on the disease has been conducted across multiple disciplines—biomedicine, clinical medicine, public health, health economics and the social sciences. The greatest volume of literature is in biomedicine and clinical medicine, and focuses on the epidemiology, diagnosis and treatment of TB, though increasing resources have been invested in understanding the complexities of the disease from a biomedical perspective to facilitate the development of new strategies for treatment (antimycobacterial drugs and recently, host-directed therapies) and prevention (a new TB vaccine). The research in public health has focused particularly on the manner in which the dual burden imposed by TB/HIV co-infection has transformed interventions in, and attitudes to, disease and transmission, as well as the heavy economic and social impact of the co-epidemic and efforts to control it.^2–7^

According to Edington,^8^ for marginalised individuals living in constrained conditions, TB control strategies such as DOTS (*d*irectly *o*bserved *t*herapy, *s*hort-course)—the standard 6-month combination drug regimen for drug-susceptible TB disease—illuminate the complexities of the sociopolitical conditions that frame TB illness and patient-centred care. Foster *et al* 2 also argued that challenges posed by the treatment of TB reflect the disjuncture that exists between clinical expectations of cure and the lived experience of TB illness and care. Abdool Karim *et al* 4 have similarly suggested that drug-resistant TB highlights the socioeconomic constraints that shape a TB sufferer’s experiences of the disease: patients experience a restricted sense of agency and yet an overstated sense of autonomy in their illness, treatment-seeking behaviour and ability to adhere to the demanding treatment course. Experiences of drug-resistant TB also reveal institutional deficiencies which impose a requirement to negotiate ethics, human rights and public health issues.^9^

In the social sciences, research on TB has focused on stigma, the challenges of adherence (and non-adherence) to treatment, the TB-HIV syndemic and the complexities of TB control.^10–15^ This has highlighted the entanglement of the biological and the social which is superimposed on an equally complex political economy of health and disease both locally and on the global stage.^16^ This convergence of biology, culture and politics makes TB a compelling subject through which to explore the relationship between biomedical knowledge and the understanding and experience acquired through being afflicted with or affected by an infectious disease.

Eh!woza is a public engagement programme built on a collaboration between TB scientists, a South African conceptual artist, a group of young learners and members of a TB-burdened community. The initiative grew out of the need to interrogate the relationship between the biomedical and social realities of TB; the project’s founders recognised that interactions between those who conduct research on the disease in the basic sciences and those afflicted with—or affected by—TB were limited. The intention was to provide an opportunity for young people living in TB-burdened communities such as Khayelitsha, a township on the outskirts of Cape Town with extremely high rates of TB and HIV, to interact with the biomedical researchers and to share personal as well as their communities’ understandings of TB. It was also viewed as an opportunity to expose biomedical researchers, many of whom are exclusively laboratory-based in contrast to their clinical colleagues, to the complex social and economic aspects of the disease: to shift the perspective from the pristine laboratory to the realities of patients with TB.^17^

This article examines the intersection of science, art, youth education and lay narratives within Eh!woza. It explores the ways in which the project provides young people with a platform to interrogate individual understandings of infectious disease and subsequently, the precarious construction of knowledge about TB. The article draws on the experiences of participants of the Eh!woza project and the narratives of community members with whom the young people interacted, and provides insight into how the project mediates different ways of knowing TB. We provide an analysis of two emergent themes: first, how knowledge was communicated and miscommunicated through the use of medicoscientific discourse in the set of science workshops and, second, how concepts of cure presented during the science workshops are confronted by ideas of care presented through reflections of people’s individual experiences with TB. The paper concludes by proposing that Eh!woza and the collaborations it enables represent an emerging example of medical humanities in Africa.

## Methods

The research was conducted in three sites in Cape Town between May 2016 and December 2017. The first was a research institute at a local university which conducts advanced scientific and clinical research into infectious diseases including TB and HIV. The second site was the township of Khayelitsha, and the third was media workshops in which the young people participated and produced documentary films held at the collaborating artist’s studio in Cape Town.

Khayelitsha, an overpopulated example of apartheid-era forced removals, is a periurban township on the outskirts of Cape Town that consists mainly of informal settlements and housing. The demographic is a predominantly black population, and the township is shaped by high rates of, poverty, unemployment, housing insecurity, crime, HIV and other clinical infectious diseases. It is also burdened with very high rates of TB disease, and most of the young learners participating in the Eh!woza project have themselves previously suffered from, or know a family member or someone else who has suffered from, TB.

This article draws mainly from the series of workshops that form the core of the project. There are two distinct but interlinked components to the workshop programme—science workshops and media workshops. Over a period of six Saturday afternoons, students averaging between the ages of 15 years and 17 years participate in a series of science workshops facilitated by biomedical research groups based at the IDM. This is followed by an intensive 2-week media workshop held at the collaborating artist’s studio during the winter school holidays. Students participate interactively and create short documentaries to interrogate social issues associated with infectious diseases within communities of their choice.

Data were collected through focus group discussions, participant observation, in-depth and semistructured interviews, and formal and informal conversations with the students, TB scientists and community members over the course of the workshops. The latter were individuals living in informal settlements within the township who had previously suffered from TB or had indirectly been affected by the disease. Interviews were conducted in English with the TB scientists while a combination of English and isiXhosa was used with students and community members. Throughout fieldwork, the majority of data were collected by working intimately with the students: documenting and conversing as they worked with the TB scientists, and by following and participating in their collecting of video footage and interviews, through the production of their documentary films, and engaging with them and their lives during their participation in the Eh!woza.

Written informed consent was given by all adult participants as well as assent from adolescents and consent from their primary guardians. There were no financial incentives for participation.

### Vignette 1: an introduction to TB science

One of the science workshops hosted by the TB biomedical researchers required that the students swab and smear bacterial samples from various surfaces of their bodies and leave them to incubate. In the follow-up workshop a week later, Joelle, a scientist, gave the students an opportunity to see what they had grown under the microscope in a different part of the laboratory housing various pieces of laboratory equipment including a microscope, flow cytometer and freezers that stored a variety of samples.

Before they looked into the microscope, Joelle described and demonstrated a technique called ‘gram staining’, commonly used to classify bacteria. She began by smearing a small sample of bacterial cells from one of the students onto a glass slide and added a crystal violet dye to the sample. The students stood around watching and listening. Joelle incubated the sample, rinsed it with water to remove the dye, added iodine to fix the crystal violet to the cell wall of the bacteria. She rinsed the sample with acetone, rinsed with water again, then added a secondary stain, and rinsed one last time. She explained that if the bacterial sample retained the crystal violet stain, it would be classified as Gram-positive and would appear purple under the microscope. If not, it would be Gram-negative and retain the secondary stain which would appear red under the microscope.

The students queued up to view their samples and record what they could see. Xolani laughed loudly as he peered into a plate containing bacteria swabbed from Aphiwe’s mouth. The bacterial colonies, as he described to one of the scientists, were ‘small, round-shaped, and plentiful’. Zandisile’s plate had bacteria that also grew in small round-shaped clusters which she proudly described to her group. Adjusting the knobs on the side of the microscope to see better, Zandisile continuously remarked: "I didn’t know that this was growing on me".

As each student marvelled at the kinds of bacteria that they had grown, they didn’t realise that they were being engaged on the biological matter of microbiology and infectious diseases and how these appeared under the microscope. This was their introduction to the practices of TB mycobacteriology.

### Encounters in the laboratory

The annual Eh!woza programme begins with a structured curriculum comprising a set of workshops that introduces scientific research and biomedical practice followed by an exploration of the complex world of mycobacteriology. An initial workshop defines the concept of bacteria and research, while those that follow describe different aspects of TB and infectious diseases more broadly. This series of workshops is supported by intermittent experiments within the host institute’s laboratories in which the students are introduced to the technical aspects of scientific research. The students dress in stained lab coats and wear blue gloves, and learn the techniques, practices and language of scientific research, moving in and out of the lab as they are introduced to the processes involved in establishing scientific knowledge.

The workshops are facilitated by TB research scientists from various biomedical fields including immunology, mycobacteriology and clinical research. Together, they guide the students through the processes involved in each experiment, evaluating the students’ grasp of core concepts and understanding of the spaces, techniques and tools with which they are working. Discussions about the life-cycle of infection and ideas around vaccination are presented by an immunology researcher, details of TB transmission are presented by an expert clinician, and the intricacies of drug discovery and antibiotic TB treatment are presented by a professor in mycobacteriology, all working to show the significance of these biological processes in understanding and curing what has been defined, through the workshops, as a biomedical problem. In this way, the science workshops provide an example of a process of inclusion between a community of scientists and a community of non-scientists: the scientists invite students into the laboratory space and students develop lines of inquiry during discussions within the workshops. The students are also offered the opportunity to assimilate these teachings for themselves.

In one of the final workshops, the students are tasked with designing research projects around different aspects of the disease based on the knowledge and understanding gained through scientific interactions in the laboratory and in the classroom. One group of students proposed a project on the efficacy of traditional medicine in treating an infectious disease such as TB. This sparked a discussion about aspects of TB treatment, particularly from students who had previously suffered from the disease and had made use of traditional medicine during their treatment-seeking. While they all agreed that biomedicine had proven efficacy and was more reliable, as reiterated in health information shared with them in school and reinforced in the recent workshops, the students discussed the influence of cultural beliefs in decisions about where to seek treatment. Some students emphasised that many people in Khayelitsha do not understand the aetiology of TB and therefore its treatment. Others spoke about the perceptions of clinics and of TB illness as deterrents to seeking treatment. For example, one student explained that some people don’t trust clinics or have easy access to them. Another explained that there is a stigma associated with TB disease and treatment.

The scientists presented a series of questions exploring what the students may have thought of the use of traditional medicine *versus* biomedicine, asking ’how would you ensure that you are not putting people at risk by telling them to treat TB with traditional medicine instead of antibiotic drugs that we know work?’ This highlighted the difficulties consequent on interspersing scientific research practices with the students’ conceptions, understandings and perceptions of different aspects of TB in their communities. In these instances, the students expressed a curiosity about, and knowledge of, different aspects of TB disease, sharing opinions with each other, drawing from their own local understandings and experiences, and building on knowledge gained during workshops.

The biomedical researchers used the workshops to communicate complex aspects of TB research and the science that drives it, as well as to translate difficult biomedical concepts and practices around scientific inquiry into a digestible form. Students spoke of and understood TB through drawing their understanding from personal experiences with the disease, from friends and family, and from health information to which they had previously been exposed. One of the students, Buhle, commented, “I just knew that (TB) was a disease that existed and that was curable after 6 months. I knew of it because of my brother’s experience with it". Another student stated that her knowledge of the disease came from school where they were only taught about symptoms such as coughing up blood and that the disease manifested in the lungs.

In one workshop session, a question posed by Joelle—‘what causes TB?’—prompted the response that smoking, alcohol abuse, pollution and poverty were some of the causes. According to Aphiwe, ‘people who smoke a lot or live in poor areas or dirty areas get TB’. Zandisile, who lives in an informal settlement in the township, explained that many people in her community were affected by the disease because of the poverty in her area. The student’s responses reflected how they understood the disease through their everyday experiences.

By inviting access, inclusion and communication, the workshops begin to demystify each group’s practices, perceptions and knowledge. When asked about their experiences within the science workshops, one group of students explained that their understanding of the disease was enhanced through their interactions with the facilitators (TB researchers and clinical experts).

They explained their work/the science in a manner that we were kind of able to understand because I didn’t even know that it was possible for me to also have TB (the bacteria) in my body without getting sick because of the fact that the soldiers (immune system) in my body are suppressing it so that it doesn’t escape. And that if I got more exposed to it, then the soldiers in my body would get weak and that’s how the TB would spread (Buhle).

In the above quote, Buhle shared her understanding of active and latent TB infection gained from the workshops. Other students presented contrasting understandings and experiences of the science workshops and the medicoscientific discourse shared.

I wish there was someone within the workshops that would take time to explain that these words mean this and that, in a way that we would be able to understand…sometimes we’re just not brave enough to ask (for clarification) (Nomusa).

I found the content of the workshops so complicated. I didn’t understand/grasp a lot of the concepts that they were telling us. If it were up to me, I would have rather spent all my time in the lab, in our lab coats (laughs). I remember Joelle giving us a presentation with all these difficult words…I can probably make sense of it through my own understanding but I don’t know the names of all the bacteria…what I found interesting was the part where we were testing (in the lab)…workings with the plates and the bacteria…it helped me understand that this is how bacteria for TB grows and is spread and that it is everywhere’ (Zandisile).

The workshops aimed at being interactive. Initially, though, the presentations by the contributing experts were intimidating; the students hesitated to contribute to the conversations and to ask questions about the information shared with them, the complex concepts which were introduced and the different aspects of the biology of TB that were presented. Lectures on drug efficacy, drug resistance and antibiotics, and concepts such as ‘minimum inhibitory concentration’ were often met with silence and nervous looks from the students who didn’t always understand the terminology or content or were too scared to voice their lack of understanding. The laboratory demonstrations, however, were liberating, offering a more engaging space that allowed the science to be translated. There, the learners began to ask questions about concepts with which they had initially struggled; the scientists and the students could engage more informally, in conversation and in practice, about what it meant to apply the concepts that they had just learnt. It was only in the laboratory demonstrations that the students began to gain a better understanding of the science through the tasks they performed and the interactions those permitted. The workshops therefore created spaces in which meanings and understandings were constantly and interactively negotiated.

### Vignette 2: living with multiple epidemics

In a recent Eh!woza programme, one group of students spent an afternoon in the neighbourhood of Makhaza in Khayelitsha. They chose to create a film about Jabulile, an HIV-positive mother in her 30s who had previously suffered from TB. At the time of filming, Jabulile had recently suffered two strokes that affected mobility on the right side of her body and had been attending rehabilitative treatment at the local clinic. The interview began with reflections from Jabulile’s mother on how she came to terms with her daughter’s experiences with illness, from the time of her infection with HIV to her most recent stroke. Speaking in a soft voice, she expressed the support that she continuously provided to her daughter. The conversation quickly shifted to Jabulile’s personal recollections of her experiences and, guided by the questions the students asked, Jabulile provided a detailed account of how each episode of illness affected her life. When the students asked questions about the kinds of treatment she was on, she stood up to fetch a small bag in which she kept all her medication. As she spoke about her HIV treatment and how it had become an important part of her daily life, she removed multiple containers from the bag and described what each bottle was for and how she took the medication.

Moments later, she suggested that everyone take a trip with her to her old house to visit family members who could provide better insight into her illness experiences and how she had managed to survive. Navigating the very narrow gaps between the shacks, the party was greeted by many people who knew Jabulile well. Two family members were seated outside her old home. Though startled by the camera, they were happy to see Jabulile, welcoming everyone in and agreeing to share their stories of Jabulile and her struggles. Their descriptions were detailed and, at many moments, very emotional with each family member expressing how Jabulile’s illness affected them personally, spiritually and economically.

In their accounts, they spoke intermittently of their own illnesses, both having suffered from TB and managing their HIV seropositivity. Jabulile’s aunt gave an account of Jabulile’s and her own experiences of getting tested and explained that Jabulile had consulted her before getting tested for HIV and that her response had been:

Wait…don’t get tested without knowing that you are sick (without symptoms). Just wait…don’t create this stress for yourself. I was the one that was being treated at that time (for HIV). Then a couple of months went by and I don’t know what happened. Then we both went to the clinic for her to get tested. She got tested and found out that she was (long pause).

Jabulile’s aunts spoke about their afflictions in code, sometimes speaking in whispers, aware of the stigma around both diseases (HIV and TB), possibly fearing being overheard by neighbours as the students recorded, never referring to HIV or antiretroviral (ARV) treatment but suggestively hinting at what was meant. The accounts given by Jabulile’s friends, gathered at a tavern nearby, were the same with one friend stating that ’we would never stress her with asking what she (Jabulile) was sick from or why she was sick. We didn’t talk about those things. We just knew that she was sick’. The relatives framed their family life with accounts of mental illness, the passing of numerous other relatives, and the secrecy and stigma that shrouded their afflictions and their daily socioeconomic struggles. Crowding the small space of the veranda with their presence and camera equipment, the students spent the afternoon listening to poignant recollections of how illnesses had profoundly changed the family members’ lives, significantly affecting how they understood themselves, and how their lived reality shaped their understanding of their illnesses. At the end of our visit, Jabulile, together with one of her aunts, shared some pictures from an old photo album containing images from before their illnesses, repeatedly stating, ‘We used to be so pretty'.

Similar stories emerged in interviews with other community members, revealing how common experiences of TB illnesses were in various communities within the township, and the different ways in which individuals negotiated the realities of infectious diseases combined with the struggles of daily life. One person, Phindi, described having a CD4 count (cluster of differention 4 - a glycoprotein found on the surface of immune cells) of 20 when she was diagnosed with TB and having to endure both TB and HIV treatment. She experienced multiple episodes of drug-susceptible and drug-resistant TB illness and attended numerous visits to her local clinic to receive injectable TB drugs. She recounted how these episodes, prolonged treatment and side effects had shaped her experience of the disease.

By that time, HIV was something that was hidden, but I still took it lightly. That is when TB attacked me. And by the time TB attacked me, my CD4 count was four (4) or zero (0)…very low. But I still had some weight on me though. It was the TB and now this and there were so many pills. And they told me that I would need to stop taking my ARV pills and for 2 weeks I would need to be taking my pills for TB and then I would return to the clinic. But I had to treat them both and treat the TB for 6 months until completion. I struggled because TB required me to have fatty foods, to eat, to have fruits like oranges. But I managed to defeat it. When I finished having TB, I had HIV to deal with on the other hand and I was dealing with the pills for HIV and I was continuing with them.

My daily basis I spent at the clinic, I never defaulted. Then I got TB for the second time and I asked the nurses ‘how’s that possible?’ because I treated it and I finished my 6 months treatment. And they told me that my environment is dirty because at that time they told me that TB was in the air because people cough and spit so we catch it, especially if your body’s soldiers (immune system) are low. And at that point I didn’t know that we have blood cells… I learnt that blood cells were those things that were in your immune system. I treated it and finished it and it took 8 months with that painful needle.

The third time I was like ‘hell no! I can’t have TB for the third time’ and they told me ‘sis Phindi, your sputum still shows that you have TB and you still have to go on with your treatment’ and I had to obey the rules to go on with the TB treatment, until I finish it. Where I am now, I’m here today. I survived. (Phindi)

### Encounters in the field

After a series of weeks engaging with the science behind TB, the Eh!woza programme shifts into media workshops, moving away from the laboratory into the field and the studio. The students are introduced to video recording equipment and tasked with collecting footage representing as well as reflecting perceptions of the disease—their own, those of the communities of choice, in and around their township, constructing stories that characterise their personal understanding of TB. The youth are encouraged to drive the content of films and directions of the interviews, and the interaction within the community usually begins by asking: ‘What do people know about TB?’. Through interrogating this question, the students document a variety of (mis)conceptions about symptoms and risk factors, ideas about contagion and disease transmission. More importantly, the students uncover and document the illness testimonies that community members present to explain personal experiences of the infectious disease.

In describing her illness history, Jabulile began with her HIV seropositivity, moving on to provide a recollection of her stroke and repeated experiences with TB illness. Through these accounts, she described her initial diagnosis of TB at a local clinic, her introduction to anti-TB treatment and her struggles with adjusting to its side effects:

My first time taking the medication, I had a problem that whenever I was drinking the pills, I would throw up. But then you have to take them again because it is important. You can’t stay without taking the pills. After vomiting, I would drink the medication again because it was important for me to have the pills in my body (Jabulile).

Jabulile suffered a stroke that led to loss of employment and later experienced a second episode with TB which revealed TB infection in her intestines. Though visiting the clinic on numerous occasions, she struggled to get diagnosed:

I had no idea what it was because I visited the clinic two times and they were unable to find anything. They made me give sputum samples and they were negative. And then they put me through the X-rays and in the X-rays that they put me through they couldn’t see anything in my body. And they told me that they didn’t have any other way to test me. But I continued to feel weak and that was the time before I went to the hospital…(I was drinking) a mixture of small pink tablets like those for the heart and other white ones too…almost 13 pills a day. This treatment took me 6 months and it ended in October last year…but now I’m back to just taking my ARVs (Jabulile).

Through these recorded narratives and testimonies of illness, young people participating in the project make use of visual elements to reflect themselves as members of a TB-burdened community and what this means for them. Their participation in Eh!woza in turn enables the viewer to see how the youth come to terms with the realities of an infectious disease endemic within their communities, the biological complexities of the disease and the illness narratives of community members. Asking students how they come to understand peoples’ experiences with TB in their communities through Eh!woza’s media workshops, we received the following responses:

When we did our work in Nkanini, everyone in Nkanini could relate to the problem (of TB). They’d say this person has TB or that person has TB. And with the film we did with Phindi she had TB three times living in the township of Nkanini, so that’s what people were showing us. We wanted this to be seen by a lot of people and for them to understand the severity of it (TB) and go to the clinics. And then the nurses could also take time to go out of the clinic and talk to people (Zandisile).

Through my experience, I saw that the people that are most affected are those that have HIV…especially with their weaker immune systems that make death more threatening if they don’t take their treatment (Owami).

(even though the threat of TB infection is constant), what I learnt was that despite this, people seem to know how to take care of each other during times of illness. They know how to help each other. Just like that woman we met in TR Section (an informal settlemnet in Khayelitsha) that had TB of the spine, she said that there were people that helped her. People have a sense of community (Ntombizodwa).

The films also provide insight into how the students engage with ideas of mortality and risk, the sick body, conceptions of treatment and cure, and experiences of social suffering. Buhle’s group made a film which documented the experience of her brother Themba, who had suffered from TB meningitis. Recollections shared by her family members described how Themba disclosed his TB diagnosis and his HIV status at the height of his illness. Through the film, Buhle stated that her brother used to hide his medication underneath his mattress and pretend to his family that he had taken all his treatment. The story explored his TB symptoms, the challenges of TB/HIV co-infection, how the side effects affected his ability to commit to treatment and his abuse of alcohol during his sickness. The film describes profound changes that occurred in his body throughout his sickness from weight loss to the loss of his eyesight, a possible insight into the mental health of a suffering person, through to his eventual passing.

Since Jabulile’s film, other groups have reproduced this now-established style of reflecting the lived experiences of people affected by TB. The content of the films has therefore changed over the 4 years of the project: the initial films comprised miscellaneous interviews with multiple community members, whereas recent efforts have been in-depth stories about an individual who has been affected by TB and associated factors. As interviewees describe the impact of the disease on their lives, the viewer is provided with insight into the lived realities of community members. By interspersing these interviews with scenes from science workshops, the films highlight that, in South Africa, TB—a biomedical problem in that it is caused by a bacterium and can be treated by antibiotics—is inextricably linked to the social conditions of the communities it affects.

Through this, we see the contribution of the young students to Eh!woza as crucial. As a generation, their recognition within, and reflections on, their communities are often marginalised. One group of students enunciated the lack of youth visibility within their community, stating that, ‘although we are kind of visible, as youth we want to be more visible’. Another student, Zinzi, commented:

This is how they recognise youth…like I wouldn’t say in a positive way, because they’re like okay there are young people and they party, they do drugs, all these things. That’s how we’re being seen…we are the trouble-makers that parents need to deal with…it’s only the negative things like the crime that we do or the drugs or teenage pregnancy (Zinzi).

Another group observed that, as young people in their communities, they are denied platforms from which they can highlight and address issues collectively from the youth’s perspective. They lack people or programmes that can provide them with knowledge, and the means or opportunities to share and make use of it among their peers. This breeds a sense of apathy about the things that affect them and their communities.

Reflecting on their participation in Eh!woza, one of the students, Unoluthando, said:

What was even more enticing was the fact that young people were being given a chance to go in, to do things that they were not supposed to be doing, things that are not common for young people to do/to be doing. Because like when I go out there and explain to people that I have a film that I made on my own, that I edited and did everything myself, people don’t believe me because this thing is not something that young people are used to doing/that is common for young people to do. So I feel like I’m special or I’m different because I was able to get the/a chance from Eh!woza to allow me to be a part of it/join so that me and my friends could do this thing (Unoluthando).

Another explained that, to students like herself, a project like Eh!woza was an opportunity for young people to present different issues that they could address collectively:

The way that we understand and interpret things is different. I feel that we can change people’s perspectives. I feel like it’s easy for young people to change the ways in which people see things. I think we can…and we can show things from a different perspective for people…we can present things to people from the way that we understand them’ (Nonkululeko).

Two emergent themes were uncovered during encounters in the laboratory and in the field, and these are analysed in the remainder of this paper.

### Disputed knowledge: the communication and miscommunication of knowledge through the science workshops

Latour and Wolfgar’s^18^ *Laboratory Life* provides a reflection on what the science workshops revealed to be an invitation into the lab. Observing and exploring the routine practices of scientific work within a laboratory, the authors examine what scientists do in their spaces and the way scientific facts are constructed therein. They argue that the work of the laboratory and its scientists consists of a series of interactions and transformations at the laboratory bench involving a range of samples, substances and apparatus that eventually produce particular kinds of evidence and establish the networks in which this evidence or facts are made meaningful.^18^

In Eh!woza, the community of scientists give the youth access to their world and expose students to the specimens, techniques and technologies that shape their work as laboratory-based TB researchers. The project presents an opportunity to see how knowledge about the disease is established through the scientific practices, the community of TB researchers who navigate, negotiate and participate in these practices, what they produce, and how they come to present this to a group of young people from a TB-burdened setting as a particular way of knowing the disease. The science workshops engage young people on the biological realities of TB disease and mediate particular kinds of ‘knowing and the concepts that they present provide a particular way of ‘seeing', as noted in the writings of Good:^19^ biomedicine and its practices ‘(construct) persons, patients, bodies, diseases and human physiology’ in a particular manner that constitutes a particular reality and relation to the world.

The workshops also present specific ideas about how illness and the body are constructed through the practices of biomedical TB research; these shape how the young people involved in the project come to know TB. By explaining the tools involved in TB research, the various technologies integrated into this exploration of the disease and the therapeutic activities, the workshops configure the body and understandings of TB in a specific way that calls attention to its material nature, its physiological facts and its molecular processes.^20^ These form a certain way of seeing and understanding the disease, a particular kind of reality, which ‘formulates sickness in strikingly materialist terms’.^19^

The workshops thus enable an evaluation of the mystique of science and how it defines knowledge about and engagement with disease and conceptions of illness. Much like Emily Martin’s^21^ work on the scientific models and medical metaphors that govern knowledge, particularly about women’s bodies and their experiences, the science workshops and interactions therein also present opportunities to find ‘alternative visions’ and ‘other ways that (infectious diseases) could be described’. In this way, opportunities are provided to deconstruct the ways of knowing an infectious disease such as TB that are dominated by biomedical knowledge and scientific practices, imagery and models, medical metaphors and descriptions. In some respects, therefore, the science workshops require young people from TB-burdened communities to make sense of this knowledge, juxtaposing biology and culture through their participation.

Rapp,^22^ writing about the communication of amniocentesis testing through genetic counselling, explores the ways that information about the working of scientific technologies is mediated and shared with a community of non-scientists: scientific information is communicated and miscommunicated so that ‘meanings are actively and interactively produced’ by the experts and laypersons, ‘revealing and creating some meanings that mask or silence others’.^22^ Rapp highlights ‘the vocabulary of biomedicine’, and the cultural contexts in which this information is communicated and interpreted.^22^ This knowledge is not always successfully translated or easy to interpret, revealing the tensions therein. In the Eh!woza workshops, the students tried to find ways to make sense of the new knowledge; their lessons present ‘statistics and medical terminology (as) genres of communication, not simply neutral vocabularies’.^22^ In bringing light to this concept, Rapp shows that those who are invited into this medicoscientific discourse—the Eh!woza students in this case—are not passive recipients of information but respond to it in different ways, both succeeding or struggling to translate the knowledge shared with them.

Robins^23^ offers a reflection on the production of knowledge around AIDS in local communities in rural South Africa through prevention and treatment programmes. In this work, he highlights how AIDS activists presented global biomedical interventions through ’translating and mediating biomedical ideas and practices into vernacular forms that (could) be easily understood and acted on by the ‘targets’ of these recruitment strategies’ sharing the possibilities of AIDS science and medicine as new biomedical technologies of health citizenship.^23^ While observing and participating in the project, the science workshops and laboratory experiments provide a stimulating and interactive platform for engaging with the biomedical conceptions of TB, and allow us to follow the emerging conversations which, on the one hand, foreground the socioeconomic realities and local knowledge that shape experiences of the disease in high-burdened communities (as presented by the students) and, on the other, how this intersects with the biology of the disease (as presented by the scientists). Building on concepts such as drug susceptibility and resistance, or immune responses to infectious pathogens, the project’s workshops strive to ‘vernacularize’ biomedical concepts and mediate a particular kind of knowledge about TB. As shown above, the ways in which the students participate in, or make sense of, this ‘vernacularization’, reveal the complexity of the workshop’s medicoscientific discourse and the knowledge being shared. They also highlight the necessity of these conversations and attempts to translate the technical and biomedical aspects behind TB science and the local perceptions and knowledge that shape people’s understandings of the disease.

### Cure versus care: young people confronting biomedical knowledge and engaging with the social experience of TB illness through film

The vignette about Jabulile and the corresponding film exemplify the manner in which the Eh!woza project and its incorporation of documentaries reveal narratives of TB illness from affected community members. These testimonies are punctuated with references to CD4 counts, HIV medication, loss of employment, regular visits to the clinic for anti-TB treatment, and daily life shaped by treatment and adherence.

Documenting these narratives, the young people in the project bear witness to social suffering. The way the youth choose to frame these experiences becomes an act of social justice by making visible the injustices ensured by the TB-affected. But, they also help us interrogate the differences between concepts of cure that focus on the biomedical aspects of illness (as presented in the science workshops) and ideas of care expressed in the narratives and experiences of affected community members that emerge through candid interviews conducted in different neighbourhoods within the township.

Through Jabulile’s and similar stories, what is highlighted is the contrasting ways in which experiences of TB are articulated. The science workshops present TB through understanding of the biology of the disease and biomedicine’s response to it in the form of antibiotic treatment and cure. In contrast, the media workshops, through the narratives and descriptions presented by community members, highlight how their experiences with the disease are shaped by unemployment, stigma and misconceptions, socioeconomic constraints, family support or lack thereof, and how all these aspects inform the ways in which they are able to navigate, negotiate and leverage care throughout these experiences.

## Eh!woza as a medical humanities project

Eh!woza highlights and interrogates the relationship (or lack thereof) between biomedical knowledge and practices and the social experiences, conceptions and narrations of illness, how these are interpreted by young people through art and film, and how the films examine and reflect the experiences of people affected by disease. It facilitates an exchange of ideas and experiences between the biomedical researchers, youth and community participants. In its embrace of interdisciplinarity, the project offers an opportunity to present and draw on different perspectives and voices to engage with the textured experiences of ill-health in a TB-burdened community. It is a project about both access to knowledge and diverse ways of knowing: the science workshops and the scientists who facilitate them present biomedical understandings of TB as infectious disease; the media workshops, the artist who facilitates them, the participating students, the community members they interact with, and the use of visual media and film, present young people’s perspectives and community members’ local understandings and social experience of the same infectious disease, punctuated by narratives of TB illness.

Levine^24^ writes that understandings of illness cannot be reduced to what is reflected under the microscope and that there is a need in (bio)medicine to see beyond this. In the same vein, Lock and Nguyen^16^ present a discussion of how medical knowledge should be understood as deeply social, existing within an intersection of social, cultural and political contexts, ‘an assemblage of knowledge and practices inextricably associated with political expediencies, social interests, and embedded values.’ Young,^25^ writing on the anthropologies of illness and sickness in the early 1980s, expresses a similar and still relevant idea that ‘the task at hand is not to simply demystify knowledge, but to critically examine the social conditions of knowledge production’ and how everyday experiences of health, illness, and healing are shaped and transformed through this.

The influences of North American and British medical humanities introduced ideas of integrating (bio)medicine and art to emphasise the human aspect of medical practice in an interdisciplinary and multidisciplinary manner that both fosters collaboration and dialogue around issues of health.^26^ Eh!woza, and projects similar to it, provide a means to explore a medical humanities initiative driven by local cultural, political, historical and geographical contexts. Drawing from both the science and the social and through practice and conversation, it enables an exchange of ideas about health and illness between scientists and youth and members of communities with high rates of TB. In many respects, for those involved, it provides an embodiment of transformation and an act of social justice. In a country where the majority of TB sufferers are black, poor and vulnerable to ill-health and those wearing lab coats are white, well off and healthy, a project such as Eh!woza makes it clear that illness is not merely accidental—social context, environment and circumstances shape the bodies we have; and the bodies we have shape our experiences of and in the world.
